# A Histological and Clinical Evaluation of Long-Term Outcomes of Bovine Bone-Derived Xenografts in Oral Surgery: A Systematic Review

**DOI:** 10.3390/jfb16090321

**Published:** 2025-09-01

**Authors:** Angelo Michele Inchingolo, Grazia Marinelli, Irma Trilli, Gaetano Del Vecchio, Angela Di Noia, Francesco Inchingolo, Massimo Del Fabbro, Andrea Palermo, Alessio Danilo Inchingolo, Gianna Dipalma

**Affiliations:** 1 Department of Interdisciplinary Medicine, University of Bari “Aldo Moro”, 70124 Bari, Italy; angeloinchingolo@gmail.com (A.M.I.); graziamarinelli@live.it (G.M.); trilliirma@gmail.com (I.T.); dr.gdelvecchio@gmail.com (G.D.V.); angeladinoia@libero.it (A.D.N.); gianna.dipalma@uniba.it (G.D.); 2 Department of Biomedical, Surgical and Dental Sciences, Milan University, 20122 Milan, Italy; massimo.delfabbro@unimi.it; 3 Unit of Maxillo-Facial Surgery and Dentistry, Fondazione IRCCS Ca’ Granda Ospedale Maggiore Policlinico, 20122 Milan, Italy; 4 Department of Experimental Medicine, University of Salento, 73100 Lecce, Italy; andrea.palermo@unisalento.it

**Keywords:** Bio-Oss, bovine-derived xenograft, bovine bone graft, xenograft, bone regeneration, guided bone regeneration, GBR, histological evaluation, clinical outcomes, complications, long-term, follow-up

## Abstract

Background: Bovine bone-derived xenografts are widely used in regenerative dental procedures due to their osteoconductive properties and volumetric stability. However, their long-term behavior and biological integration remain a subject of debate. This systematic review aims to critically assess the histological and clinical outcomes associated with bovine xenografts over extended follow-up periods. Methods: An electronic search was performed in PubMed, Scopus, and Web of Science, including studies published in the English language from 2005 to 2025 for a total of 217 records, which were initially identified from PubMed, Scopus, and Wos. Results: After duplicate removal, following title/abstract screening and full-text evaluation, 11 studies met the inclusion criteria. These studies reported on the use of bovine-derived xenografts in clinical contexts, assessing parameters such as graft integration, histological remodeling, complication incidence (e.g., chronic inflammation or foreign body reactions), and implant success rates over time. Conclusions: The current evidence suggests that bovine-derived xenografts, particularly Bio-Oss^®^, are associated with favorable long-term outcomes in bone regenerative procedures, demonstrating satisfactory graft integration and implant survival rates. However, variations in study design, follow-up duration, and outcome measures warrant further high-quality, long-term randomized clinical trials to confirm these findings and guide clinical decision-making.

## 1. Introduction

**Figure 1:**
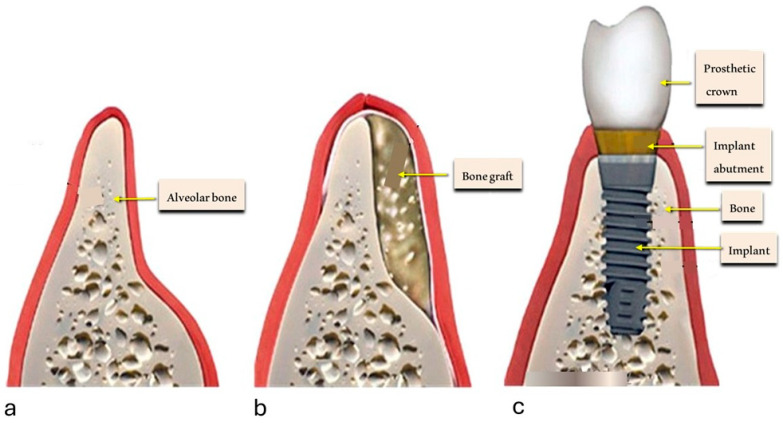
Schematic representation of the bone augmentation process using bovine bone-derived xenografts. From left to right: (**a**) resorbed alveolar ridge with insufficient bone volume; (**b**) application of bone graft material to augment the deficient area; (**c**) final stage showing successful implant placement with restored bone volume, implant integration, and prosthetic crown. This sequence illustrates the clinical rationale for xenograft use in implant site development prior to prosthetic rehabilitation.

Esthetics and functionality are essential components of contemporary dental treatment [1,2,3,4]. However, the predictability of clinical outcomes, reliability of therapeutic protocols, and particularly long-term prognosis continue to pose significant challenges [5,6,7,8,9]. The ultimate goal of dental treatment extends beyond short-term success and should include long-term biological stability [10,11,12,13]. In this context, regenerative bone procedures represent one of the most delicate areas where a critical risk-benefit assessment is warranted [14,15,16]. Over the past decades, there has been a significant increase in dental implant surgeries that require regenerative techniques such as guided bone regeneration (GBR) or sinus augmentation [17,18,19,20]. Among the biomaterials employed, bovine-derived xenografts have become the most frequently used substitutes due to their osteoconductive properties and availability (Figure 1) [21,22,23,24].

Products such as Bio-Oss^®^ are widely used in clinical practice, mainly due to their reported safety profile and slow turnover, reflecting their persistence and gradual integration within host bone [25,26,27,28]. Nonetheless, emerging clinical and experimental data challenge this notion, raising concerns about the actual biodegradability and long-term behavior of these materials [29,30,31,32,33,34]. Despite their widespread usage, only a limited number of scientific studies have systematically analyzed the adverse effects and long-term complications associated with bovine xenografts [35,36,37,38]. While many studies highlight their volumetric stability and histological compatibility, fewer reports systematically address potential delayed complications such as foreign body reactions, chronic inflammation, or material migration [39,40]. A critical concern lies in the human host’s limited capacity to degrade and replace these xenograft particles [41,42,43,44,45,46]. Unlike autogenous or synthetic grafts, bovine-derived bone substitutes often resist biological breakdown. Phagocytosis, one of the primary immune mechanisms for clearing mineral debris, appears to be ineffective against the microstructure of xenograft particles [47,48,49,50]. As a result, these materials can remain intact for extended periods, potentially becoming a source of chronic inflammation or acting as foreign bodies [51,52,53,54,55]. Recent clinical cases and animal studies have provided evidence supporting this concern. Seok et al., for example, demonstrated through histological and X-ray microanalysis the potential for xenograft particle migration from the surgical site to distant anatomical locations [56,57,58,59,60]. These findings suggest that xenograft materials may not always behave as passive fillers, and their persistence or displacement could contribute to pathological processes [61,62,63,64,65]. Reported clinical complications include acute and chronic maxillary sinusitis, material displacement, foreign body reactions, encapsulation, chronic inflammation, and soft tissue fenestration. Concerns regarding potential disease transmission, although mitigated by current manufacturing processes, remain under discussion [66,67,68,69,70]. Although current manufacturing processes claim to eliminate prion infectivity, the extremely long latency period of such diseases (potentially decades) leaves the door open for long-term concerns that may remain undetected in the early postoperative years [71,72,73,74,75,76]. Moreover, many studies that support the use of bovine xenografts suffer from significant methodological heterogeneity [77,78,79,80,81]. Variability in study design, inconsistent definitions of clinical success, and lack of long-term follow-up data limit the generalizability and reliability of the conclusions drawn [82,83,84]. Some reviews report favorable outcomes without addressing essential variables such as particle size, resorption rate, or immunologic compatibility [85,86,87,88,89,90,91,92,93]. In many cases, the resolution of clinical complications associated with xenografts is achieved only through surgical removal of the migrated or encapsulated material, a procedure that demands precise diagnosis, high surgical skill, and carries inherent risks [92,93,94,95,96].

Despite the large body of literature on the clinical use of bovine-derived xenografts, there is a clear gap in evidence regarding their long-term biological behavior and possible delayed complications. Most existing reviews have focused primarily on short-term outcomes such as volume stability or early implant survival, while systematic assessments of adverse events, material migration, and chronic inflammatory reactions are scarce. The novelty and relevance of the present systematic review lie in its focus on identifying and synthesizing data about long-term safety concerns, an area that has been underrepresented in previous analyses. By addressing this gap, our review aims to provide clinicians with a more balanced understanding of the risk–benefit profile of bovine xenografts.

The purpose of the present report is to raise awareness about the long-term risks and possible late-onset complications associated with the use of bovine-derived xenografts in oral surgery [97,98,99,100,101]. Drawing from clinical observations, case reports, and a critical review of the literature, we aim to provide a comprehensive overview of the potential pitfalls of relying solely on short-term clinical success [102,103,104,105,106,107]. Immediate functional or esthetic outcomes do not necessarily correlate with long-term biological integration or patient safety [108,109,110,111,112,113]. This discussion is not intended to dismiss the utility of bovine xenografts entirely but to emphasize the need for a more cautious, evidence-based approach in their application [114,115,116]. As regenerative procedures become increasingly common, the dental community must prioritize long-term outcomes and patient safety over convenience or tradition [117,118,119,120,121,122]. Further studies, particularly those involving long-term follow-up and standardized assessment protocols, are essential to better understand the biological behavior and clinical implications of bovine-derived graft materials.

In light of these considerations, the primary objective of this systematic review is to evaluate the evidence regarding the long-term safety and biological behavior of bovine-derived xenografts in dentistry. The secondary objectives are to (i) identify and describe the reported late-onset complications associated with their use, (ii) analyze the methodological limitations of the existing literature, and (iii) highlight areas requiring further research in order to guide future clinical practice.

In conclusion, while bovine xenografts have demonstrated utility in bone regeneration procedures, their use should be critically evaluated on a case-by-case basis, especially in light of emerging evidence of complications.

## 2. Materials and Methods

In this paper, we conducted a systematic review following the Preferred Reporting Items for Systematic Reviews and Meta-Analyses (PRISMA 2020 [123]) guidelines using the PubMed, Web of Sciences (WOS), and Scopus databases with the PROSPERO code CRD420251111685.

### 2.1. PICO Question

Population (P): Patients undergoing dental implant surgery requiring bone regeneration.

Intervention (I): Use of bovine-derived xenografts (e.g., Bio-Oss^®^) for guided bone regeneration or sinus augmentation.

Comparison (C): Alternative grafting materials or techniques (e.g., autologous bone, porcine xenografts, synthetic substitutes, or platelet concentrates).

Outcome (O): Long-term clinical and histological outcomes, including graft integration, complication rates (e.g., chronic inflammation, graft migration, foreign body reaction), and implant success over time.

Focused PICO question:

In patients receiving regenerative bone procedures for implant therapy, does the use of bovine-derived xenografts—compared to other grafting materials—affect long-term clinical and histological outcomes, including complication rates and implant survival?

### 2.2. Search Processing

An electronic search was performed in PubMed, Scopus, and Web of Science, including studies published in the English language from 2005 to 2025. The search strategy incorporated combinations of keywords such as “Bio-Oss”, “bovine-derived xenograft”, “bovine bone graft”, “xenograft”, “bone regeneration”, “guided bone regeneration”, “GBR”, “histological evaluation”, “clinical outcomes”, “complications”, “long-term”, “follow-up”, combined using Boolean operators (AND, OR) to maximize the sensitivity and specificity of the results.

### 2.3. Inclusion Criteria

Studies were included if they met the following criteria: (1) the use of bovine-derived xenografts (e.g., Bio-Oss^®^) for guided bone regeneration or sinus augmentation in vivo, (2) available as open-access publications, (3) written in English (including cases where only the abstract was in English), and (4) focused on adult populations.

### 2.4. Exclusion Criteria

The exclusion criteria encompassed: (1) meta-analyses, (2) animal studies, (3) in vitro research, (4) review articles, (5) studies unrelated to the topic, and (6) no free full text.

The references of the included literature were thoroughly examined to identify additional papers that could be relevant.

Two authors (I.T. and G.D.V.) performed the article extraction according to the inclusion criteria, and any disagreement was resolved after adequate discussion and comparison.

### 2.5. GRADE Assessment

To further assess the strength and quality of the available evidence, we applied the GRADE (Grading of Recommendations Assessment, Development, and Evaluation) framework. Each included study was evaluated across five domains: risk of bias, inconsistency, indirectness, imprecision, and publication bias. Evidence from randomized controlled trials (RCTs) was initially rated as high quality, while observational studies started as low quality. Downgrading or upgrading was applied depending on methodological limitations, sample size, follow-up duration, and robustness of outcome assessment.

The overall certainty of evidence for each primary outcome (graft integration, histological remodeling, complication incidence, and implant survival) was graded as high, moderate, low, or very low. This structured approach enabled a transparent evaluation of the reliability of the findings and informed the strength of clinical recommendations derived from this review.

## 3. Results

**Figure 2:**
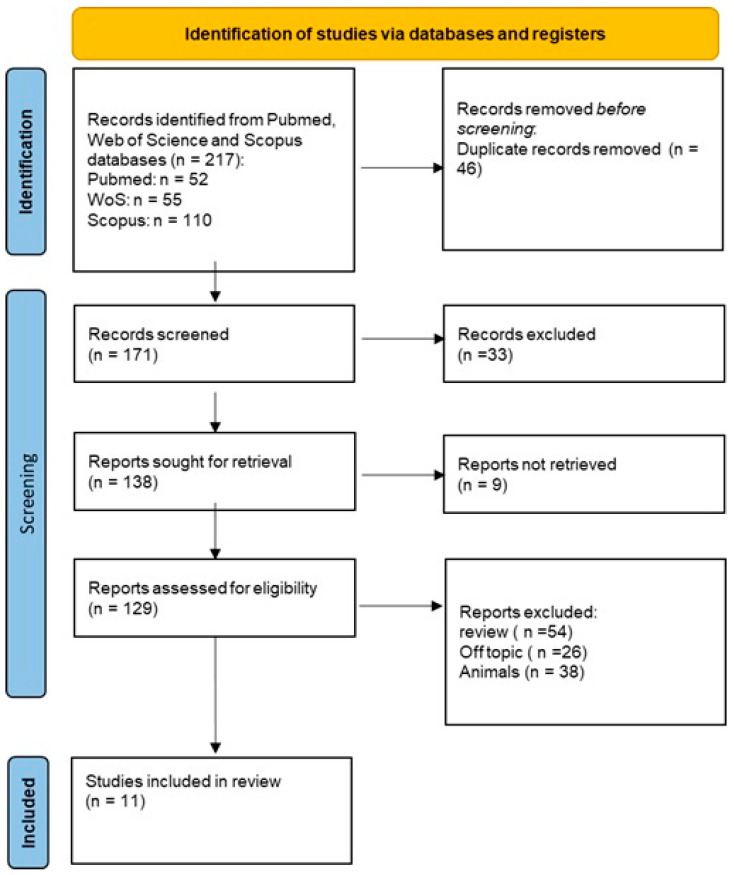
PRISMA flow diagram.

Initially, a total of 217 documents were identified through the literature search: 52 from PubMed, 55 from Web of Science (WOS), and 110 from Scopus. After removing 46 duplicate articles, 171 articles were assessed. Among these, 33 studies did not meet the inclusion criteria, and 9 of them were not retrieved. Subsequently, we screened for eligibility and inclusion criteria, resulting in the inclusion of 11 papers. The study process and PRISMA flowchart are summarized in Figure 2, while detailed summaries of the included articles can be found in Table 1.

### 3.1. GRADE Assessment of Evidence

Using the GRADE approach, the overall certainty of the evidence was variable across outcomes. For implant survival with bovine xenografts, the evidence was rated as moderate quality: randomized controlled trials consistently reported high survival rates, although heterogeneity in follow-up duration and reporting reduced certainty. Evidence regarding histological remodeling and graft integration ranged from low to moderate quality, as some RCTs provided robust biopsy data, but variability in methods and relatively short follow-up periods limited confidence. For long-term complications such as graft migration, chronic inflammation, and sinus pathology, the certainty was judged as low, being mainly derived from case series and observational data with potential underreporting. Finally, comparisons with autografts or porcine grafts were supported by moderate quality evidence, with RCTs indicating similar short- to mid-term outcomes, though long-term data remain insufficient.

### 3.2. Risk of Bias Assessment

The methodological quality of the 11 included studies was evaluated using the ROBINS-I tool (Risk Of Bias In Non-randomized Studies—of Interventions), which assesses seven domains: bias due to confounding, selection of participants, classification of interventions, deviations from intended interventions, missing data, measurement of outcomes, and selection of the reported result. The findings are summarized in Table 2. Overall, most studies demonstrated a moderate risk of bias, particularly in the domains of confounding and missing data. These were often affected by incomplete follow-up, absence of control groups, or unadjusted baseline differences. For example, studies by Barone (2005), Kim (2025), Lai (2020), and Rodriguez (2019) showed multiple domains rated as moderate risk, especially in areas most susceptible to methodological variability [125,131,132,134]. Three studies—Couso-Queiruga (2023), Schmitt (2013), and Hashemipoor (2020)—presented missing or unreported data in one or more domains, highlighting partial methodological transparency [128,130]. Among all included studies, Mordenfeld (2017) was the only one rated as low risk across all seven domains, representing a methodologically robust reference point [127]. The study by Huang (2025), despite an overall low risk profile, showed a critical risk in the domain of deviations from intended interventions. This likely reflects the challenges in standardizing an innovative hydraulic technique during surgery and quantifying intraoperative variables [124]. Studies by Nowzari (2022) and Rodriguez (2019), which focused on long-term complications of bovine-derived xenografts, exhibited moderate risk across multiple domains, reflecting the inherent limitations of observational and case series designs and variability in outcome assessment protocols [125,126]. It is important to note that randomized controlled trials were excluded from this specific ROBINS-I visual representation to maintain methodological consistency within the evaluated cohort. This assessment underscores the need for greater methodological rigor, including prospective designs, effective control of confounding variables, longer follow-up periods, and comprehensive histological validation. Transparent reporting and consistent outcome measures are essential for reliably evaluating the long-term safety and performance of biomaterials in implant dentistry.

To enhance clinical applicability, we summarized the practical implications of the findings in Table 3, which provides evidence-based clinical recommendations for the use of bovine-derived xenografts in implant dentistry.

## 4. Discussion

Maxillary sinus floor elevation and alveolar ridge preservation (ARP) are two pivotal procedures in modern implant dentistry. With continuous advancements in biomaterials and surgical techniques, clinicians now have a wide range of options, including xenografts, autografts, allografts, synthetic materials, and autologous platelet concentrates. Despite the wide application of these techniques, their long-term effectiveness, safety, and biological compatibility remain topics of considerable debate. This comprehensive discussion integrates the findings of eleven relevant clinical trials and case studies to provide a thorough and logically structured evaluation of the current clinical landscape.

### 4.1. Innovative Techniques: Hydraulic Approach and A-PRF

The 2025 study by Huang et al. introduced a novel hydraulic sinus floor elevation technique, utilizing advanced platelet-rich fibrin (A-PRF) [124]. Based on Pascal’s principle, the technique provides a minimally invasive method to lift the Schneiderian membrane, significantly reducing the risk of perforation, which is a common intraoperative complication. The application of controlled hydraulic pressure using an incompressible fluid allowed uniform lifting, with no intraoperative membrane perforations reported. Moreover, the study demonstrated that A-PRF achieved a mean bone gain of 4.05 mm at 6 months, slightly higher than previous reports using conventional PRF. A-PRF, with its denser fibrin structure and higher content of growth factors, showed a superior osteogenic potential compared to deproteinized bovine bone mineral (DBBM). These outcomes were also associated with an improved postoperative profile, including reduced infection rates due to the antimicrobial properties of leukocyte-rich A-PRF. However, the authors noted several limitations: variability in the biological quality of PRF due to donor factors, challenges in intraoperative quantification, and imaging limitations that hindered precise post-op graft height evaluation. While A-PRF shows promise, further studies with larger sample sizes and histological verification are needed to confirm these findings [124]. Two case series by Rodriguez and Nowzari, in 2019 and in 2022, critically examined the long-term risks associated with bovine-derived xenografts. Complications, often manifesting 2–13 years postoperatively, included graft migration, chronic inflammation, oroantral communication, foreign body reactions, cyst formation, and sinus infections. Histologic evidence confirmed the persistence of residual graft particles surrounded by inflammatory cells, suggesting poor biointegration. Furthermore, concerns about the complete deproteinization of DBBM were raised, as studies found residual bioactive proteins such as TGF-β and BMPs, which may induce immune responses. Notably, the thermal processing techniques used to sterilize these materials may not fully eliminate prion contamination risk (BSE). These findings challenge the assumption that DBBM is entirely inert and safe for long-term use [125,126]. The 2017 randomized controlled trial by Mordenfeld compared DBBM/autogenous bone mixtures at 90:10 and 60:40 ratios for lateral ridge augmentation. The 60:40 group showed lower volumetric reduction, but both mixtures supported successful implant placement. Interestingly, the proportion of DBBM did not correlate directly with improved clinical outcomes, suggesting that volumetric stability does not necessarily translate into superior osseointegration [127]. Hashemipoor (2020) further explored corticocancellous freeze-dried bone allograft (FDBA), with and without autogenous bone, for horizontal ridge augmentation. Histological analysis indicated that combining autografts with FDBA enhanced bone maturation and reduced graft resorption, reinforcing the benefit of synergistic grafting approaches. However, stabilization methods and the absence of rigid fixation may significantly influence graft behavior postoperatively [128]. In 2013, Christian Martin Schmitt conducted a comparative histological evaluation of four materials, Straumann BoneCeramic, Bio-Oss^®^, Puros^®^, and autologous bone, for sinus augmentation. Autologous bone exhibited superior osteoinductive properties and the highest proportion of newly formed vital bones. Xenografts and allografts, while demonstrating good space-maintaining capacity, had variable residual particle levels and lower integration. The findings underscore the importance of histological verification in biomaterial assessment and support the continued designation of autografts as the clinical gold standard [129].

### 4.2. Healing Time and Alveolar Ridge Preservation

Couso-Queiruga’s 2023 randomized clinical trial investigated the role of healing duration (3, 6, and 9 months) in ARP with collagenated DBBM (DBBM-C). Results demonstrated a progressive increase in mineralized tissue (from 13.5% at 3 months to 37% at 9 months) and a decline in residual graft material, indicating that longer healing times favor bone maturation, even when using low-resorbable xenografts [130]. Moreover, Lai (2020) compared bovine and porcine xenografts, both showing similar outcomes in preserving ridge volume. However, the facial aspect of the alveolus was consistently more susceptible to resorption, especially when the facial bone wall was ≤1 mm [131]. In 2025, Hyunjae Kim evaluated the combination of DBBM-C with or without free gingival grafts (FGG) in ARP. The study demonstrated that FGG improved the preservation of horizontal ridge dimensions, acting as a sealing barrier and enhancing soft tissue stability. While histologic and radiographic outcomes did not differ significantly between the groups, the additional soft tissue support provided by FGG may improve esthetic outcomes and minimize complications [134]. The landmark study by Barone (2005) explored the histological and histomorphometric outcomes of combining porcine corticospongious bone with iliac crest autografts for maxillary sinus elevation in severely atrophic cases. The 1:1 mixture leveraged the osteoinductive properties of autografts and the volume-stabilizing scaffold of porcine xenograft. Results indicated acceptable graft consolidation and early implant stability, though long-term histologic integration of the xenograft remained unclear. Cultural and religious factors were noted as potential limitations to xenograft use, particularly in regions where porcine derivatives may be unacceptable [132]. Correia (2024) conducted a three-year randomized controlled trial comparing porcine-derived collagen-retained cortico-cancellous bone with autografts in lateral sinus lift procedures. Both groups showed high implant survival and low marginal bone loss, aligning with systematic reviews. The ease of handling and lower donor site morbidity of the porcine graft present a favorable alternative, especially in cases with limited autologous bone availability [133]. Revisiting the 2013 study by Schmitt, we see a clear hierarchy in osteogenic potential: autografts outperform allografts and xenografts, which vary in performance. Bio-Oss^®^, while widely used, exhibited slow resorption and persistent residual particles, potentially affecting long-term bone vitality. This highlights the need for materials that balance scaffold function with predictable resorption kinetics [129]. Huang’s study underscored the significance of adjacent teeth in influencing sinus floor morphology and bone regeneration [124]. Teeth adjacent to the graft site contributed to a concave sinus floor profile, reducing membrane tension and supporting better graft containment and vascularization. This anatomical factor is crucial when planning sinus augmentation, especially in partially edentulous patients.

### 4.3. Implications for Long-Term Implant Success

Although most studies have reported satisfactory short-term implant survival, concerns remain regarding the long-term impact of non-resorbable xenografts. Studies by Rodriguez and Nowzari revealed delayed complications such as peri-implantitis, sinus infections, and persistent foreign body reactions. Systematic reviews confirm that such complications, although relatively infrequent, are underreported and likely underestimated in routine clinical assessments. Based on this integrated analysis, the ideal approach to bone regeneration in implant dentistry should consider not only short-term outcomes, such as volume preservation and early osseointegration, but also long-term tissue compatibility and the risk of complications.

Autogenous bone remains the gold standard due to its osteoinductive and osteogenic properties. However, its limitations—including donor site morbidity and limited availability—have spurred the exploration of alternatives. Xenografts, especially DBBM, while effective in preserving volume, raise substantial concerns regarding long-term biocompatibility and degradation. Porcine xenografts and platelet-rich fibrin products, such as A-PRF, offer promising alternatives with better resorption profiles and more favorable biological behavior. Nonetheless, robust long-term randomized clinical trials and histological evaluations are urgently needed to confirm their safety and efficacy.

Finally, the selective publication of positive findings, known as the “Proteus Phenomenon,” must be addressed. Applying the GRADE framework provided an additional layer of methodological rigor to our systematic review. Overall, the certainty of evidence was moderate for implant survival and graft integration, suggesting that current findings can guide clinical decision-making with some confidence. However, evidence regarding long-term complications, such as xenograft persistence, foreign body reactions, and sinus pathology, was graded as low, mainly due to reliance on observational studies, small sample sizes, and heterogeneity in outcome definitions. This highlights the urgent need for well-designed, multicenter RCTs with extended follow-up and standardized histological protocols. Until such evidence becomes available, recommendations regarding bovine xenografts should be made cautiously, prioritizing patient safety and transparent communication about potential long-term risks. Transparent reporting of complications and negative outcomes is essential for scientific integrity and patient safety. Only through balanced, long-term, evidence-based evaluation can clinicians make informed decisions when choosing the most appropriate biomaterial and technique for each individual case.

### 4.4. Clinical Utility of the Findings

The present review highlights that while bovine-derived xenografts are effective in maintaining ridge volume and supporting implant survival in the short to mid-term, their limited resorbability and persistence in host tissues raise relevant long-term concerns. Clinicians should therefore critically weigh the benefits of volumetric stability against the potential risks of late complications, including chronic inflammation, material migration, and the need for secondary surgical intervention. From a practical standpoint, these findings underscore the importance of transparent risk communication, comprehensive informed consent, and careful patient selection. Autogenous grafts remain the gold standard where feasible, whereas alternatives such as porcine xenografts or platelet-rich derivatives may represent safer options in selected cases. In daily clinical practice, the decision to use bovine xenografts should be individualized, balancing surgical convenience with long-term biological safety and patient expectations.

### 4.5. Future Research and Clinical Directions

Future research should aim to overcome the current limitations of heterogeneous methodologies, short follow-up periods, and insufficient histological validation. Large-scale, multicenter randomized controlled trials with long-term follow-up are essential to clarify the true biological behavior and safety profile of bovine-derived xenografts. Standardized definitions of clinical success, systematic reporting of complications, and robust histomorphometric analyses will be crucial to generate reliable evidence. Comparative trials evaluating bovine xenografts against autogenous bone, porcine-derived substitutes, and novel biomaterials should further guide evidence-based material selection. Clinically, emphasis should be placed on patient-centered outcomes, ethical considerations, and medico-legal implications. Advancing regenerative dentistry requires not only innovation in biomaterials but also a shift toward transparency, long-term monitoring, and patient safety as the primary endpoints.

## 5. Conclusions

The routine use of bovine-derived xenografts in dentistry requires re-evaluation in light of emerging long-term evidence. While effective in preserving volume and space for regeneration, their limited resorbability may lead to chronic inflammation, foreign body reactions, and material migration, challenging assumptions of long-term biocompatibility. Unlike autologous grafts, bovine substitutes resist degradation and can cause late complications, sometimes years after surgery. Theoretical risks such as prion transmission also remain, while current studies are often limited by short follow-up, methodological heterogeneity, and lack of histological validation. Reports of cases requiring secondary surgical removal highlight the clinical and ethical implications. A cautious, evidence-based approach is needed, emphasizing long-term outcomes, transparent communication, and comprehensive informed consent. Alternatives such as autologous grafts, porcine xenografts, and platelet-rich derivatives show promise but require rigorous comparative studies. Ultimately, long-term safety and sustainability of outcomes must prevail over procedural convenience.

## Figures and Tables

**Table 1:** Analysis of the study included in the discussion section.

Authors and Year	Type of the Study	Materials and Methods	Outcomes
Huang et al., 2025 [124]	Randomized Clinical Trial	40 patients with 46 implants randomized into two groups; measurements post-surgery and 12 months post-load; evaluated outcomes via CBCT.	Both groups achieved 100% implant survival, with advanced platelet-rich fibrin (A-PRF) group showing lower infection rate.
Rodriguez and Nowzari, 2019 [125]	Case Series	5 patients requiring surgical xenografts removal and reconstruction with autogenous grafts.	Post-treatment complications Resolved only after surgical removal.
Nowzari, Teoh & Rodriguez, 2022 [126]	Case Series	Bovine-derived xenograft (Bio-Oss) particles used in dental implant surgeries with or without autogenous grafts.	Intact/migrated xenograft particles years after surgery, with complications including chronic inflammation, peri-implant sulcus exposure, and soft-tissue disturbances.
Mordenfeld et al., 2017 [127]	Randomized Controlled Trial	20 patients randomized into two groups underwent lateral ridge augmentation using Deproteinized Bovine Bone Mineral (DBBM) mixed with autogenous bone, with bone biopsies collected and analyzed after 9 months.	The study found no significant differences in new bone formation, residual graft material, or connective tissue volume between groups, with the 60:40 group showing slightly less volumetric graft reduction.
Hashemipoor et al., 2020 [128]	Randomized Controlled Clinical Trial	25 patients underwent horizontal ridge augmentation using corticocancellous freeze-dried bone allograft, with CBCT and core biopsies for preoperative and 6 months ridge width assessment.	Both groups achieved implant placement, with FDBA + autogenous group showing higher bone width gain and new bone formation, and lower graft resorption, indicating enhanced stability and maturation.
Schmitt et al., 2012 [129]	Comparative Histological Study	30 patients underwent bilateral maxillary sinus augmentation with simultaneous implant placement. Four biomaterials were compared: autologous bone, Straumann^®^ BoneCeramic, Bio-Oss^®^, and Puros^®^. Bone biopsies were harvested after 6 months for histomorphometric analysis.	Autologous bone showed the highest percentage of newly formed vital bone, while biomaterials like Bio-Oss^®^ and BoneCeramic supported bone formation and implant integration.
Couso-Queiruga et al., 2023 [130]	Randomized Clinical Trial (RCT)	42 patients randomly assigned to 3 groups according to healing time after ARP with DBBM-C and collagen matrix sealing: Group A: 3 months Group B: 6 months Group C: 9 months. Evaluation of histomorphometric, clinical, digital imaging, implant-related and PROMs outcomes.	Longer healing time, mineralized tissue, non-mineralized tissue, and residual graft are observed in alveolar ridge reduction, particularly on facial bone. Implant placement is feasible, and patient discomfort and wound healing improve over time.
Lai et al., 2019 [131]	Randomized controlled clinical trial (RCT)	44 patients to receive ridge preservation with either Bio-Oss or Zcore cancellous xenograft, with histological and clinical measurements after 18–20 weeks.	The study found no significant difference in new bone formation, residual graft, or connective tissue between bovine and porcine patients, with porcine patients requiring additional grafting.
Barone et al., 2005 [132]	Randomized controlled clinical trial (RCT)	18 patients with bilateral maxillary sinus augmentation used a split-mouth design, with bone biopsies taken at 5 months post-implant placement.	A 1:1 mixture of autogenous and porcine bone is as effective as autogenous bone alone for maxillary sinus augmentation in terms of histologic and histomorphometric outcomes.
Correia et al., 2024 [133]	Randomized Controlled Trial (RCT), split-mouth, 3-year follow-up	20 patients with bilateral posterior maxillary edentulism underwent sinus lift using autologous bone graft and porcine xenograft,	The study found that xenograft with collagen is a viable alternative to autograft with a 100% implant survival rate, similar marginal bone loss, and no patient preference for one material.
Hyunjae Kim et al., 2025 [134]	Randomized Controlled Clinical Trial (RCT), single-blind, 3 arm, parallel-group	54 patients randomized into 3 groups: Control: spontaneous healing DBBM-C: deproteinized bovine bone mineral with 10% porcine collagen (Straumann XenoFlex) placed in the socket without sealing DBBM-C/FGG: same material plus socket sealing with free gingival graft (FGG) Bone biopsies, CBCT at baseline and 180 days, implant placement, histological and 3D volumetric analyses were performed.	Both DBBM-C and DBBM-C/FGG groups better preserved alveolar ridge volume than control. DBBM-C/FGG showed less horizontal ridge resorption at 1 mm (*p* = 0.049) and better volume maintenance at 3 mm (*p* = 0.026) compared to control. No significant differences in implant stability or safety issues. Socket sealing with FGG improved graft stability and bone quality.

**Table 2:** Overall risk of bias assessment in studies: analysis based on ROBINS-I.

Study	Bias due to Confounding	Selection Bias	Bias in Classification of Interventions	Bias due to Deviations from Intended Interventions	Bias due to Missing Data	Bias in Measurement of Outcomes	Bias in Selection of Reported Results	Overall Risk of Bias
Correira et al. (2024)	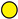	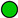	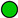	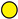	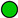	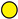	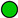	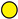
Barone et al. (2005)	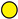	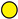	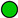	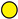	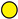	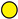	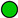	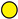
Kim et al. (2025)	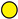	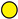	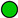	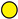	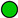	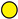	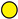	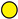
Lai et al. (2020)	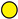	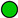	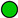	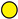	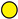	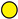	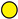	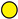
Couso-Queiruga et al. (2023)	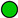	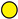	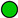	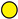	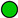	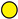	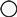	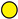
Schmitt et al. (2013)	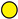	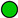	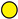	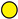	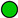	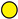	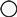	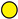
Hashemipoor et al. (2020)	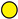	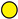	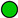	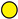	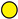	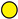	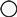	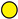
Mordenfeld et al. (2017)	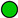	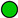	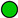	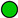	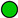	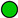	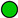	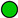
Nowzari et al. (2022)	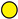	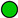	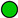	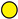	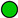	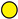	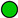	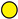
Rodriguez et al. (2019)	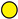	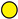	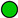	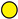	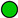	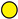	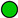	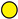
Huang et al. (2025)	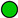	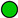	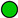	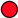	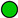	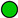	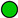	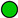

Legend: 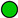 low risk; 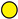 moderate risk; 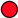 high risk; 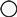 missing data.

**Table 3:** Clinical recommendations based on the systematic review findings.

Domain	Evidence Summary	Clinical Recommendation
Implant survival with bovine xenografts	Moderate-quality evidence from RCTs shows high survival, though follow-up periods are often limited.	Bovine xenografts (e.g., Bio-Oss^®^) can be safely used for short- to mid-term implant survival, but caution is advised for long-term reliance.
Histological remodeling and graft integration	Low–moderate evidence; biopsies show limited resorption and persistence of particles.	Consider that bovine xenografts provide volume stability but integrate slowly; autogenous or mixed grafts may improve biological remodeling.
Long-term complications (migration, chronic inflammation, sinusitis)	Low-quality evidence from case series; complications appear underreported.	Inform patients about potential long-term risks; monitor closely and obtain informed consent highlighting the possibility of late complications.
Comparisons with autografts/porcine grafts	Moderate-quality evidence supports comparable short-term results; porcine grafts show better resorption profile.	Use autografts where feasible (gold standard). Porcine xenografts or platelet concentrates may be preferred alternatives for improved remodeling and safety.
Risk communication & patient safety	Reports of late removal and chronic inflammation highlight medico-legal and ethical concerns.	Discuss risks transparently with patients; obtain informed consent emphasizing both benefits and limitations of bovine xenografts.

## Data Availability

No new data were created or analyzed in this study. Data sharing is not applicable to this article.

## Abbreviations

A-PRF	Advanced Platelet-Rich Fibrin
ARP	Alveolar Ridge Preservation
BMPs	Bone Morphogenetic Proteins
BSE	Bovine Spongiform Encephalopathy
CBCT	Cone Beam Computed Tomography
DBBM	Deproteinized Bovine Bone Mineral
DBBM-C	Collagenated Deproteinized Bovine Bone Mineral
FDBA	Freeze-Dried Bone Allograft
FGG	Free Gingival Graft
GBR	Guided Bone Regeneration
PRF	Platelet-Rich Fibrin
TGF-β	Transforming Growth Factor Beta
